# Editorial: Non-coding RNAs in heart failure

**DOI:** 10.3389/fcvm.2022.1016139

**Published:** 2022-08-24

**Authors:** George W. Booz, Konstantinos A. Theofilatos

**Affiliations:** ^1^Department of Pharmacology and Toxicology, University of Mississippi Medical Center, Jackson, MS, United States; ^2^British Heart Foundation Centre, King's College London, School of Cardiovascular Medicine and Sciences, London, United Kingdom

**Keywords:** cardiac remodeling, cardiac hypertrophy, fibrosis, systolic dysfunction, gene regulation

What came first the chicken or the egg? The RNA World hypothesis places RNA at the forefront, predating both DNA and protein (1). That hypothetical primordial world has evolved into a dynamic modern perspective encompassing a plethora of noncoding RNAs that actively regulate information flow from DNA to proteins. We are still at an early stage in understanding the full depths of this process and the contributions of noncoding RNAs to diseases based on both experimental data and bioinformatics (2, 3). However, this understanding could revolutionize the practice of medicine by offering novel molecular targets, as well as new therapeutic strategies. Now Frontiers in Cardiovascular Medicine presents five contributions to a special research topic that further expands our understanding of non-coding RNAs in heart failure (Figure 1).

**Figure 1:**
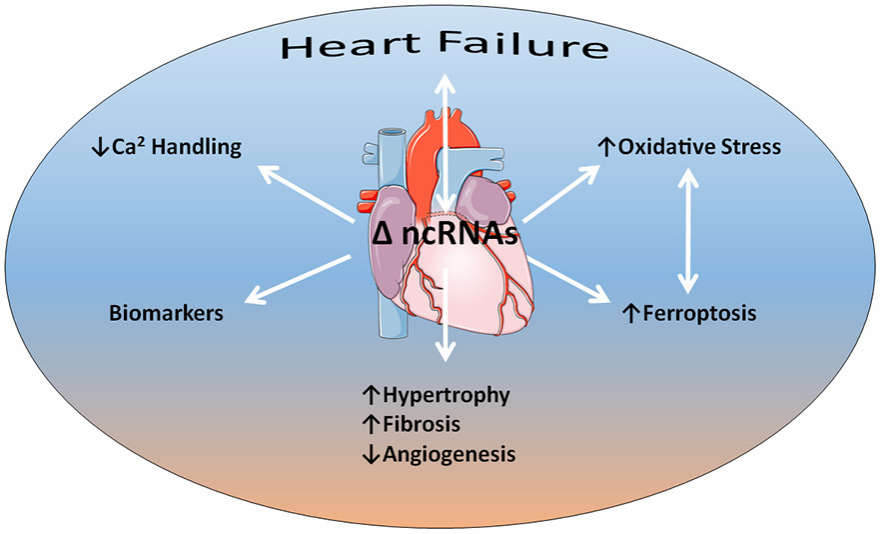
Non-coding RNAs (ncRNAs) have an intimate relationship to heart failure as both agents of cause and consequence. Several aspects of this relationship discussed in the articles of the special research topic include impairments in calcium handling and increased oxidative stress that acts synergistically with iron-related programmed cell death (ferroptosis). Evidence indicates that alterations in ncRNA expression profiles underlie adverse cardiac remodeling, such as cardiac hypertrophy, fibrosis, and capillary rarefaction or angiogenic impairment. In addition, ncRNAs may serve as biomarkers for heart failure progression. Some content is adapted from Servier Medical Art (https://smart.servier.com/) under the terms of the Creative Commons Attributions 3.0 Unported License.

Compromised cardiac function due to dysfunction in calcium handling is a hallmark of heart failure that is incompletely understood. Lei et al. examine the role played by miR-132/212, using several mouse models of heart failure and cardiac tissue from end-stage heart failure patients. They also developed miR-132/212 KO mice. Their findings indicate that the upregulation of miR-132/212 in the failing heart impairs cardiac contractile function, thereby accelerating heart failure progression. Specifically, they found that miR-132/212 decreases cardiac contractility by inhibiting expression of SERCA2, as well as by lowering its activity indirectly.

Zheng et al. explored the role of circular RNA (circRNA) in iron-related cell death or ferroptosis in heart failure. They re-constructed the circRNA-miRNA-mRNA regulatory network after firstly identifying the iron metabolism-associated genes and confirmed their findings in a mouse model of heart failure due to transverse aortic construction (TAC). Their analysis indicated that increasing levels of miR-224-5p were associated with reduced expression levels of circSnx12 and *Fth1* in heart failure with the latter being a coding gene encoding the iron binding protein ferritin heavy chain. It is thus hypothesized that circSnx12 would normally serve to sponge miR-224-5p, which in turn reduces *Fth1* expression. Ferritin heavy chain maintains iron in a safe, soluble form. Levels of increased iron, which can cause oxidative stress, and evidence of lipid peroxidation were significantly higher in the TAC group.

Morbidity and mortality from myocardial infarction (MI) remain unacceptably high due to a progression to heart failure. Wang et al. sought to identify potential long non-coding RNAs (lncRNAs) and mRNAs in the progression from acute myocardial infarction (AMI) to myocardial fibrosis to heart failure. They identified several lncRNA-co-expressed/nearby targeted mRNA pairs that may play key roles in the development of AMI, myocardial fibrosis, and heart failure.

Circulating miRNAs have gained interest as potential novel heart failure biomarkers because of sequence conservation, high stability, and ease of detection. Shen et al. performed a systematic review and meta-analysis to assess miRNA expression profiles in heart failure patients. Their study confirmed a total of 57 consistently dysregulated miRNAs related to heart failure. Moreover, their findings indicate that seven dysregulated miRNAs might be considered potential non-invasive biomarkers for HF. Additional studies are warranted to understand their specific mechanisms of action in heart failure, as well as to explore whether the combinatorial use of these miRNA markers with established HF markers, such as cardiac troponin levels, can improve diagnosis and prognosis of HF compared to existing established methods. Also, it is noteworthy that despite the ease of detection of miRNAs in biosamples with the advances of qPCR methods, there still needs to be done substantial progress to allow the measurement to be performed in an acceptable timeframe and with high enough sensitivity to allow for their application in clinical practice.

Xue et al. extensively review the role of exosomal miRNAs in heart failure. They make the case that exosomal miRNAs may be promising diagnostic and therapeutic molecules for heart failure. The processes that they may regulate include cardiac hypertrophy, fibrosis, and angiogenesis. As previously discussed, they may serve as biomarkers in heart failure to evaluate disease progression (4). In addition, the authors propose that in the future stem cell-derived exosomes may become a therapeutic strategy to treat heart failure.

Defining the role of noncoding RNAs in cardiac pathologies lies at the forefront of novel pharmacological approaches. Together with personalized medicine, this endeavor promises to revolutionize our treatment of heart failure with perhaps the goal of preventing or reversing this disease rather than simply managing the symptoms.

## Author contributions

GB and KT helped edit the text. Both authors contributed to the inception, writing of the manuscript, contributed to the article, and approved the submitted version.

## Funding

KT was supported with a BHF program grant (G/20/10387).

## Conflict of interest

The authors declare that the research was conducted in the absence of any commercial or financial relationships that could be construed as a potential conflict of interest.

## Publisher's note

All claims expressed in this article are solely those of the authors and do not necessarily represent those of their affiliated organizations, or those of the publisher, the editors and the reviewers. Any product that may be evaluated in this article, or claim that may be made by its manufacturer, is not guaranteed or endorsed by the publisher.
